# Supplementary material for: N6-methyladenosine regulates glycolysis of cancer cells through PDK4
Source: Nat Commun. 2020 May 22;11:2578. doi: 10.1038/s41467-020-16306-5 (PMC7244544; doi:10.1038/s41467-020-16306-5)
Supplement: Supplementary file 1 — Supplementary Information [file 41467_2020_16306_MOESM1_ESM.pdf]

Supplementary Information for

N^6-methyladenosine regulates glycolysis of cancer cells through PDK4

Li et al

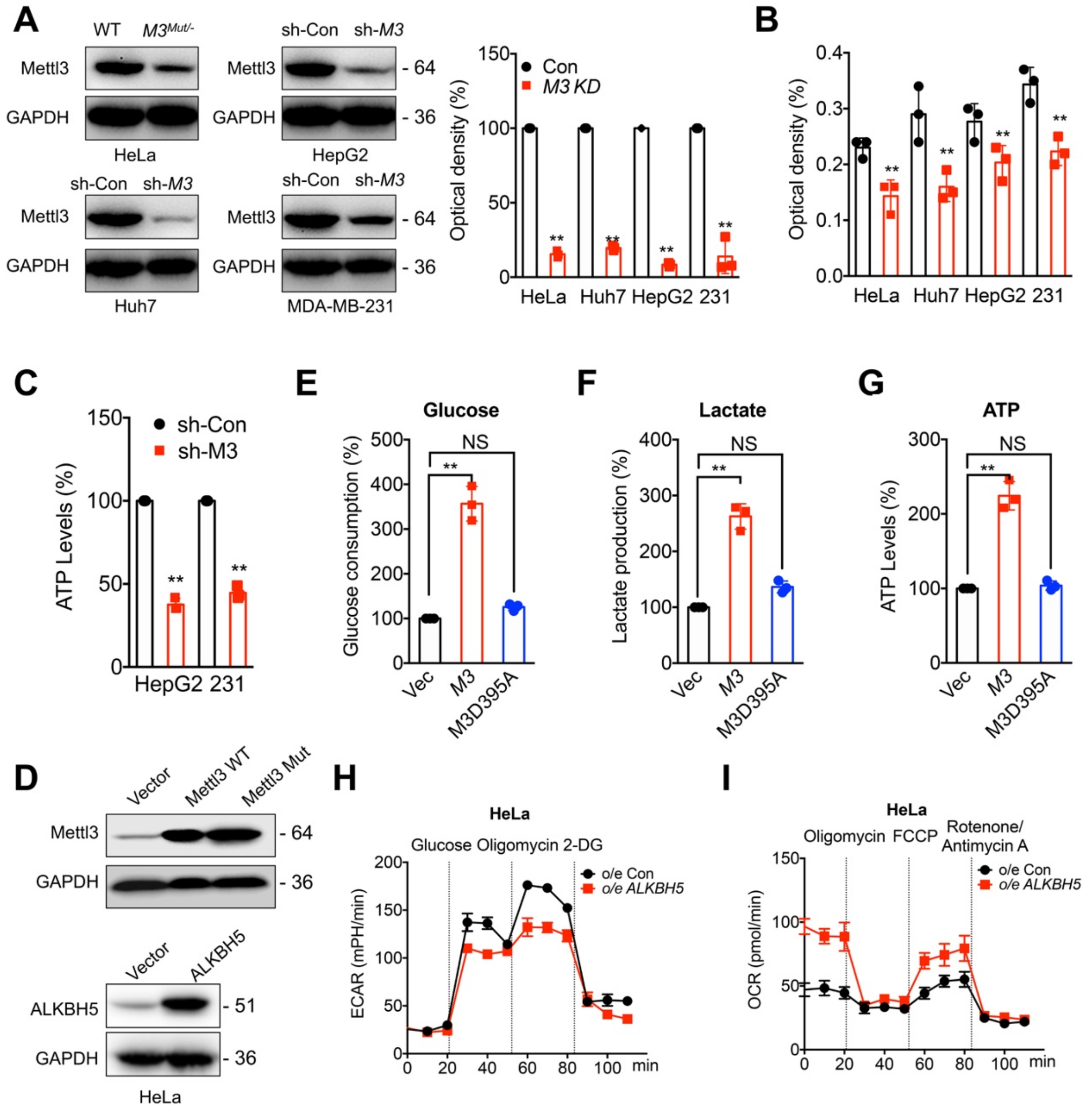

Supplementary Figure 1 m^{6}A regulates glycolysis and ATP generation of cancer cells

(A) The expression of Mettl3 in *Mettl3*$^{Mut/-}$ HeLa, sh-*Mettl3* Huh7, sh-*Mettl3* HepG2, sh-*Mettl3* MDA-MB-231 cells and their corresponding control cells were measured (*left*) and quantitatively analyzed (*right*);

(B) The m^{6}A/A ratio in *Mettl3*$^{Mut/-}$ HeLa, sh-*Mettl3* Huh7, sh-*Mettl3* HepG2, sh-*Mettl3* MDA-MB-231 cells and their corresponding control cells;

(C) The ATP levels in sh-*Mettl3* HepG2, sh-*Mettl3* MDA-MB-231 cells and their corresponding control cells;

(D) HeLa cells were transfected vector control, Mettl3 WT construct, Mettl3 DA mutant, and ALKBH5 construct for 24 h;

(E~G) The glucose consumption (E), lactate production (F), and ATP levels (G) in HeLa cells transfected with vector control, Mettl3, or Mettl3 D395A construct for 24 h;

(H&I) The cellular ECAR (H) and OCR (I) were measured in HeLa cells transfected with vector control or ALKBH5 constructs.

Data are presented as the mean ± SD from three independent experiments. A representative from a total of two or three independent experiments is shown for D. *$p<0.05$, **$p<0.01$, NS, no significant, by two-tailed unpaired Student's t-test for A ($p<0.0001$), B (p=0.011, 0.016, 0.045, and 0.006, respectively), and C ($p<0.0001$), by one-way ANOVA for E ($p=0.0003$), F ($p=0.0002$), and G ($p=0.0004$).

Related to Figure 1

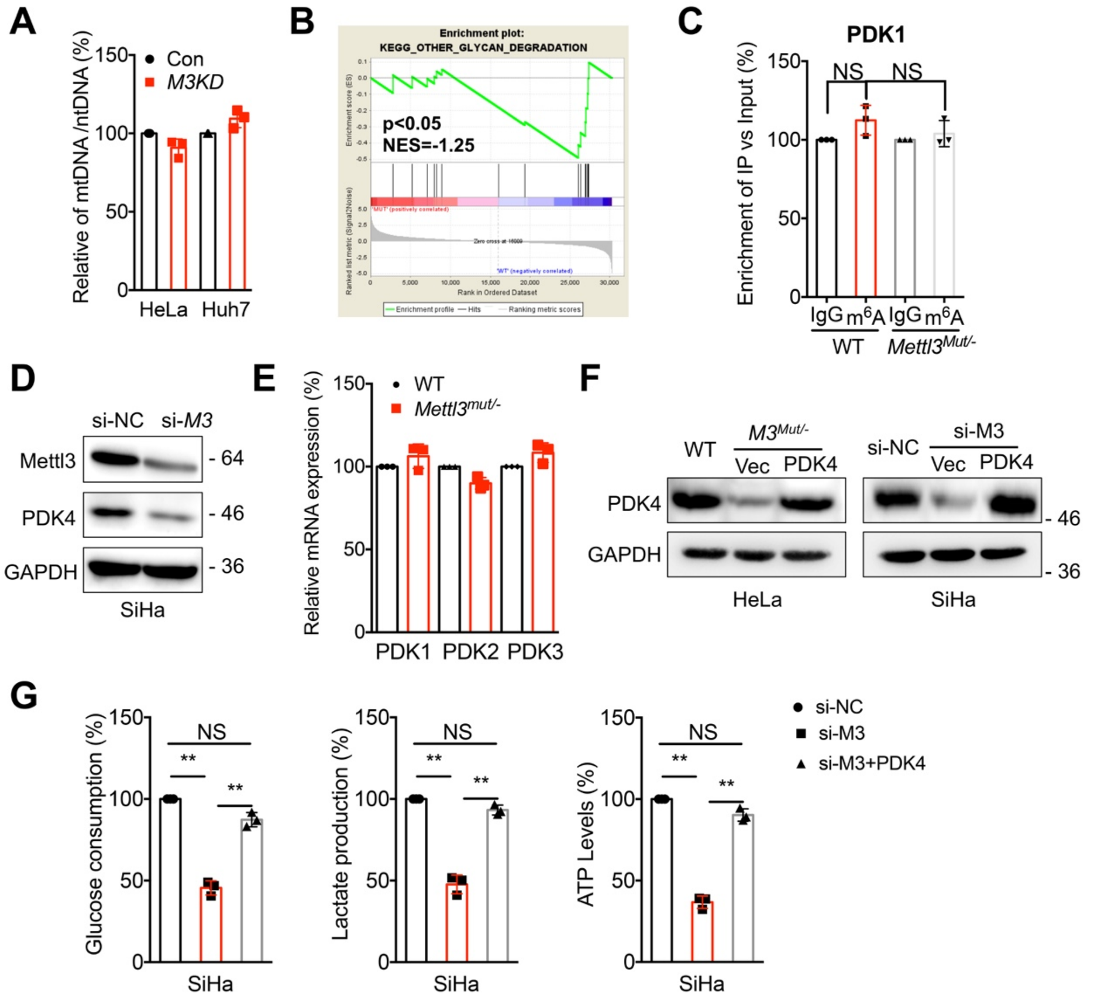

Supplementary Figure 2 PDK4 mediates m^{6}A regulated glycolysis and ATP generation of cancer cells

(A) Relative levels of mitochondrial DNA (mtDNA) to nuclear total DNA (ntDNA) were measured in *Mettl3*$^{Mut/-}$ HeLa, sh-*Mettl3* Huh7 and their corresponding control cells;

(B) GSEA revealed negative enrichment of genes in glycan degradation sets of *Mettl3*$^{Mut/-}$ HeLa cells;

(C) m^{6}A RIP-qPCR analysis of *PDK1* mRNA in wild type and *Mettl3*$^{Mut/-}$ HeLa cells;

(D) SiHa cells were transfected with siRNA for negative control (si-NC) or siRNA for *Mettl3* (si-*M3*) for 24 h, the expression of Mettl3 and PDK4 was measured;

(E) The mRNA of *PDK1/2/3* in *Mettl3*$^{Mut/-}$ and wide type HeLa cells were measured by qRT-PCR;

(F) Wild type or *Mettl3*$^{Mut/-}$ HeLa cells were transfected with PDK4 constructs for 24 h. SiHa

cells were transfected with si-NC or si-*Mettl3* combined with vector or PDK4 constructs for 24 h. The expression of PDK4 was measured;

(G) The glucose consumption, lactate production, and ATP levels in SiHa cells transfected with si-NC or si- Mettl3 combined with vector or PDK4 constructs for 24 h.

Data are presented as the mean ± SD from three independent experiments for A, C, E and G. A representative from a total of two or three independent experiments is shown for D and F. **$p<0.01$, NS, no significant, by two-tailed unpaired Student’s t-test for A and E, random permutation test for B, and one-way ANOVA for C and G ($p<0.0001$ and $p=0.0007$ for glucose consumption, $p<0.0001$ and $p=0.0003$ for lactate production, and $p<0.0001$ for ATP levels, respectively).

Related to Figure 2.

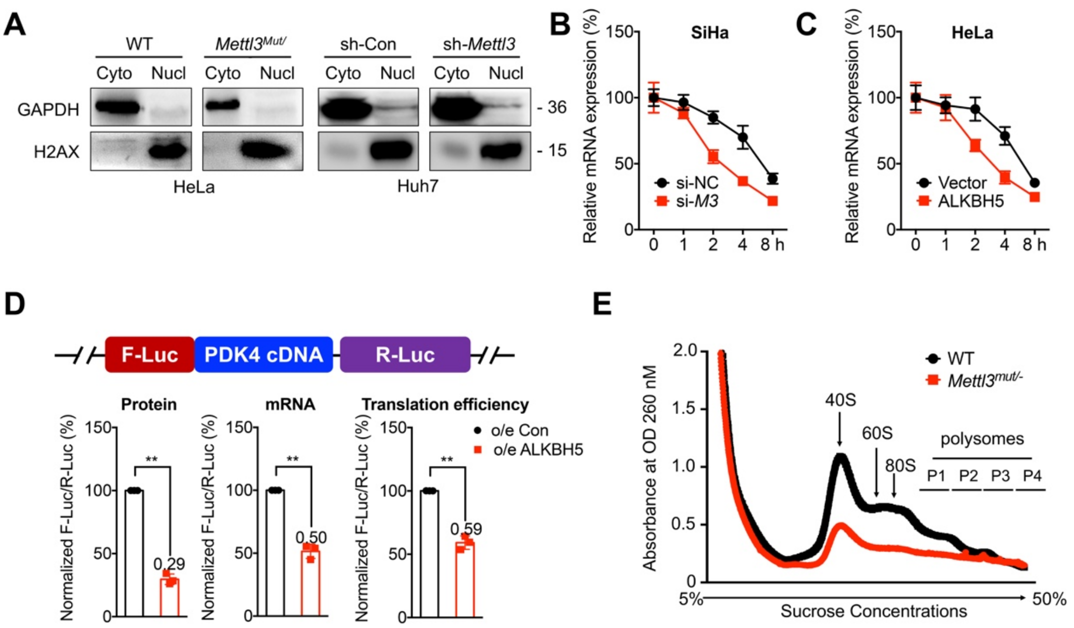

Supplementary Figure 3 m^6A regulates the mRNA stability and translation of PDK4 in cancer cells

(A) The cytoplasmic and nuclear fractions of cells were separated and checked by western blot analysis;

(B) SiHa cells were transfected with si-NC or si-Mettl3 for 24 h and then treated with Act-D for the indicated times, the mature mRNA levels of PDK4 were checked;

(C) HeLa cells were pre-transfected with vector control or ALKBH5 constructs for 24 h and then treated with Act-D for the indicated times, the mature mRNA levels of *PDK4* were checked;

(D) HeLa cells were transfected with vector control or ALKBH5 constructs combined with pmirGLO-PDK4 reporter for 24 h. The translation outcome was determined as a relative signal of F-luc divided by R-luc, the mRNA abundance was determined by qRT-PCR of F-luc and R-luc, and the translation efficiency of PDK4 is defined as the quotient of reporter protein production (F-luc/R-luc) divided by mRNA abundance [1].

(E) The polysome profiling of wild type and $Mettl3^{Mut/-}$ HeLa cells.

Data are presented as the mean ± SD from three independent experiments. A representative from a total of two or three independent experiments is shown for A. **p<0.01 by two-tailed

unpaired Student's t-test for D (p<0.0001 for all comparisons).

Related to Figure 3

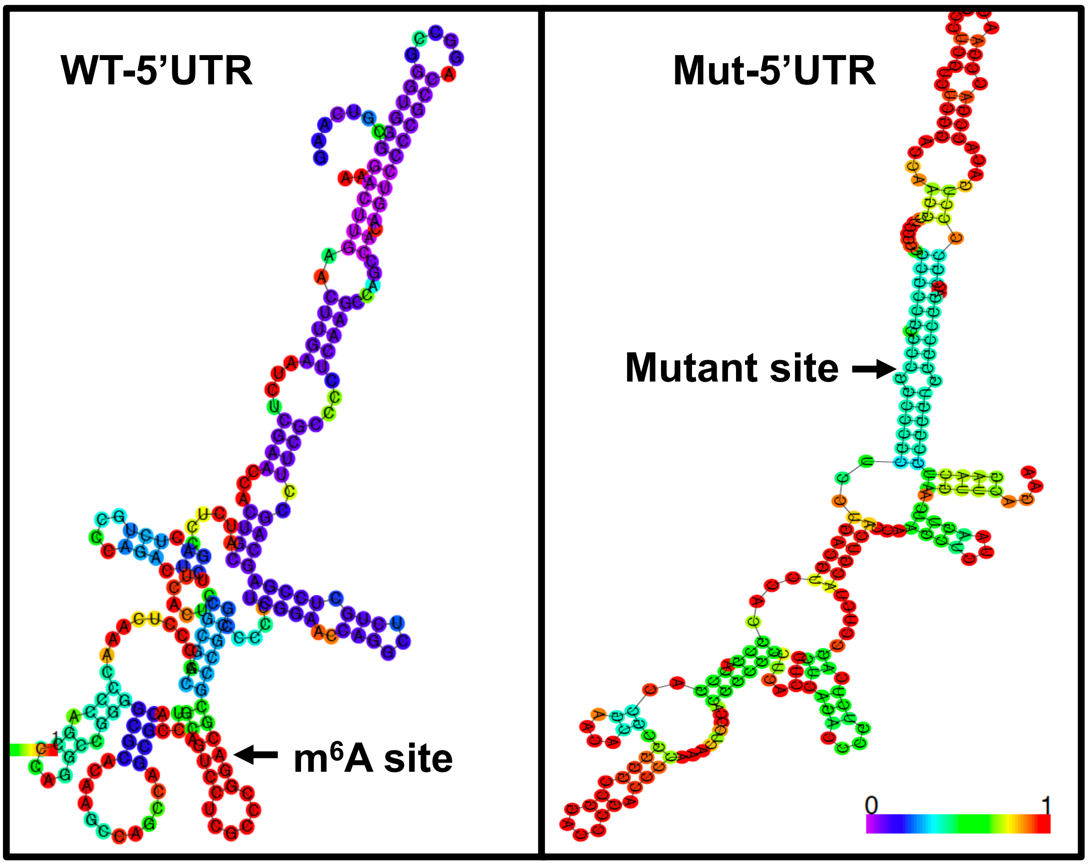

Supplementary Figure 4. The secondary structure of wild type or mutated (GG<u>A</u>C to GG<u>C</u>C) 5'UTR of *PDK4* was predicted (http://rna.tbi.univie.ac.at/). The red colour indicates strong confidence for the prediction of each base.

Related to Figure 4.

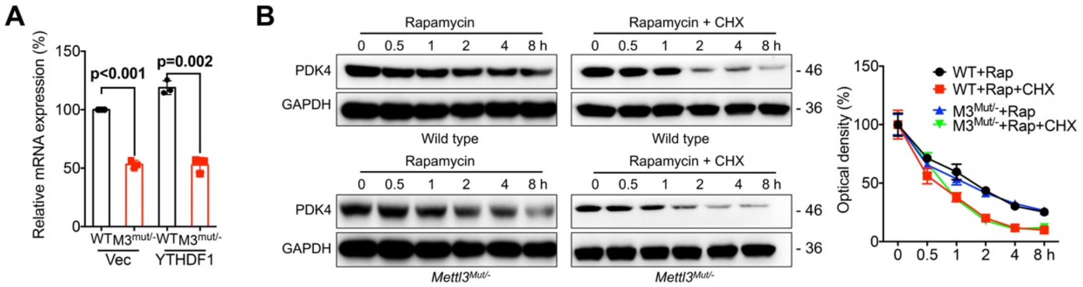

Supplementary Figure 5 Factors involved in m^{6}A regulated expression of PDK4

(A) Wild type or *Mettl3$^{Mut/-}$* HeLa cells were transfected with vector control or YTHDF1 constructs for 24 h, the mRNA expression of PDK4 was checked by qRT-PCR;

(B) Immunoblot of PDK4 expression in HeLa cells treated with 50 nM rapamycin ± cycloheximide (CHX, 10 μg/ml). A representative from a total of two independent experiments is shown.

Data are presented as means ± SD from three independent experiments and analyzed by two-tailed unpaired Student's *t*-test.

Related to Figure 5.

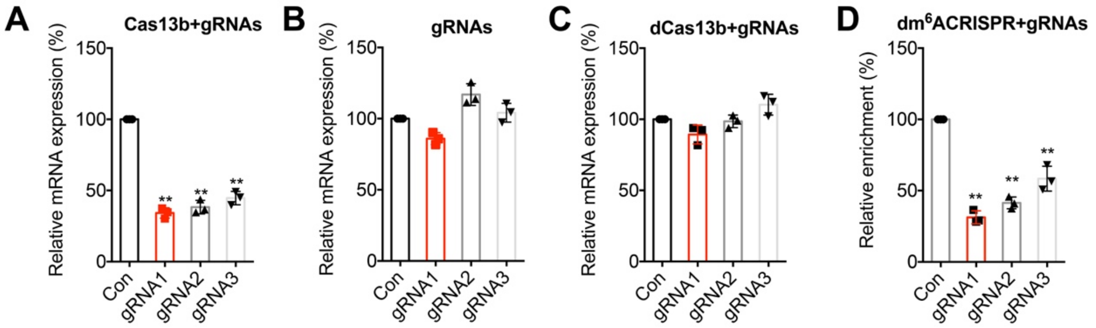

Supplementary Figure 6. Targeting m^{6}A of PDK4 by dm6ACRISPR re-programs metabolic of cancer cells

(A) The mRNA expression of *PDK4* in HeLa cells transfected with Cas13b combined with gRNA control or gRNA1/2/3, respectively, for 24 h;

(B&C) The mRNA expression of *PDK4* in HeLa cells transfected with gRNA alone (B) or combined with dCas13b (C), respectively, for 24 h;

(D) m^{6}A RIP-qPCR analysis of PDK4 mRNA in HeLa cells transfected with gRNAs and dCas13b-ALKBH5.

Data are presented as means ± SD from three independent experiments. *p<0.01 by one-way ANOVA for A (p<0.001 for all) and D (p<0.001 for all).

Related to Figure 6.

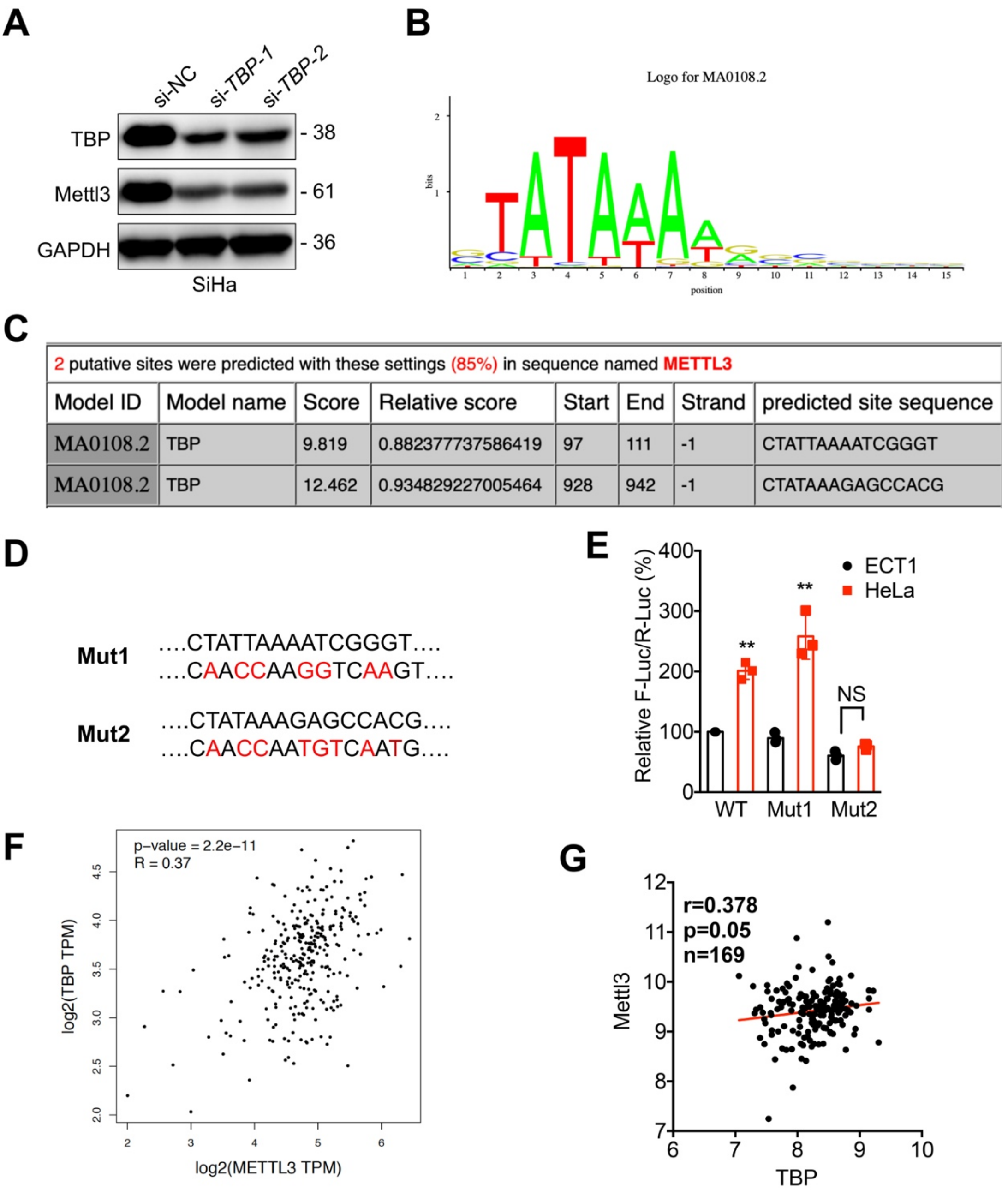

Supplementary Figure 7 TBP is responsible for the upregulation of Mettl3 in cervical cancer cells

(A) SiHa cells were transfected by siRNA negative control (si-NC) or siRNAs of TBP for 24 h, the protein of Mettl3 were checked;

(B) The binding motif of TBP analyzed by ChIPBase data;

(C) The potential binding sites of TBP in the promoter of Mettl3 were analyzed by use of JASPAR;

(D) Schematic representation of mutation in promoter to investigate the effects of TBP on

transcription of Mettl3;

(E) Cells were co-transfected with pGL3-Mettl3-WT-Luc, pGL3-Mettl3-Mut1-Luc, pGL3-Mettl3-Mut2-Luc, and pRL-TK plasmid for 24 h. Results were expressed as the ratio between the activity of the reporter plasmid and pRL-TK;

(F&G) Correlation between *Mettl3* and *TBP* in cervical cancer patients from GEPIA (n=146) and TCGA (n=169) database.

Data are presented as the mean ± SD from three independent experiments. A representative from a total of two or three independent experiments is shown for A. ***p*<0.01, NS, no significant, by two-tailed unpaired Student's t-test for E (p<0.001 for all).

Related to Figure 7.

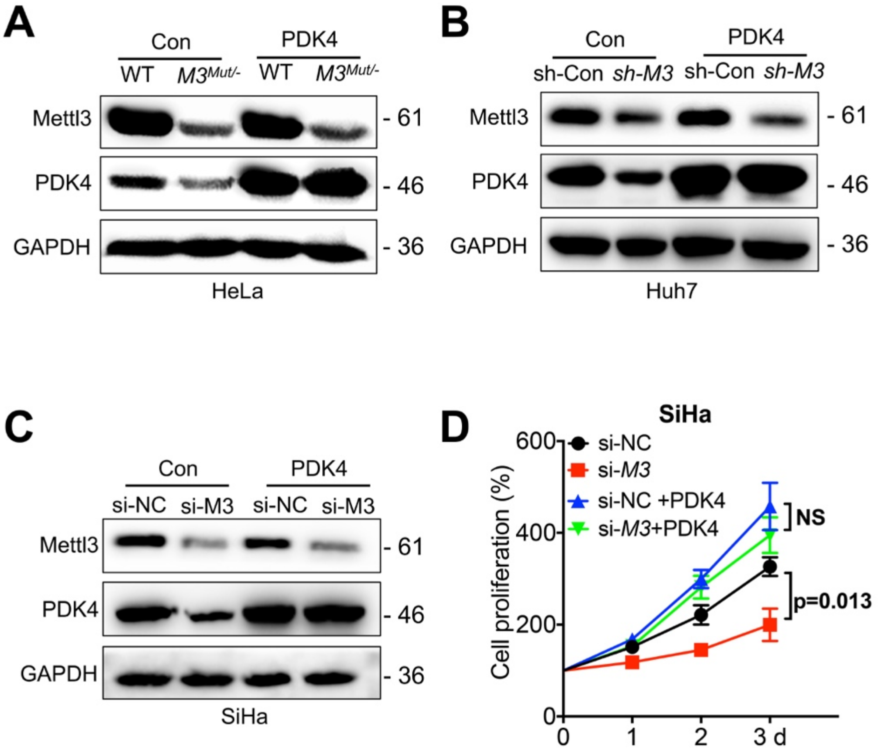

Supplementary Figure 8 PDK4 is involved in m^{6}A regulated *in vitro* and *in vivo* cancer progression

(A) The expression of PDK4 in wild type and *Mettl3*$^{Mut/-}$ HeLa cells stably transfected with vector control or PDK4 constructs were checked by western blot analysis;

(B) The expression of PDK4 in sh-control and sh-*Mettl3* Huh7 cells stably transfected with vector control or PDK4 constructs were checked by western blot analysis;

(C) The expression of PDK4 in si-NC and si-*Mettl3* SiHa cells transfected with vector control or PDK4 constructs were checked by western blot analysis;

(D) The relative cell proliferation of si-NC and si-*Mettl3* SiHa cells transfected with vector control or PDK4 constructs.

Data are presented as the mean ± SD from three independent experiments for D. A representative from a total of two or three independent experiments is shown for A, B, and C. NS, no significant, by two-way ANOVA.

Related to Figure 8.

Supplementary Table 1 The glucose metabolism-related 83 key genes

Catalog	Genes
**Glycolysis**	ALDOA, ALDOB, ALDOC, BPGM, ENO1, ENO2, ENO3, GALM, GCK, GPI, HK2, HK3, PFKL, PGAM2, PGK1, PGK2, PGM1, PGM2, PGM3, PKLR, TPI1.
**Gluconeogenesis**	FBP1, FBP2, G6PC, G6PC3, PC, PCK1, PCK2.
**Regulation of Glucose Metabolism**	PDK1, PDK2, PDK3, PDK4, PDP2, PDPR.
**Tricarboxylic Acid Cycle (TCA) Cycle**	ACLY, ACO1, ACO2, CS, DLAT, DLD, DLST, FH, IDH1, IDH2, IDH3A, IDH3B, IDH3G, MDH1, MDH1B, MDH2, OGDH, PC, PCK1, PCK2, PDHA1, PDHB, SDHA, SDHB, SDHC, SDHD, SUCLA2, SUCLG1, SUCLG2
**Pentose Phosphate Pathway**	G6PD, H6PD, PGLS, PRPS1, PRPS1L1, PRPS2, RBKS, RPE, RPIA, TALDO1, TKT
**Glycogen Synthesis**	GBE1, GYS1, GYS2, UGP2.
**Glycogen Degradation**	AGL, PGM1, PGM2, PGM3, PYGL, PYGM
**Regulation of Glycogen Metabolism**	GSK3A, GSK3B, PHKA1, PHKB, PHKG1, PHKG2

Supplementary Table 2 The overlapped genes in the Venn diagram

Names	Total	Genes
**Metabolic genes m^6A-genes mRNA-seq**	1	PDK4
**Metabolic genes m^6A-genes**	5	ACO2 IDH3G PYGL HK2 IDH3B
**m^6A-genes mRNA-seq**	65	SLC12A8 IGSF3 COL1A1 PTGES LAMB3 SAMD10 MYCL SECTM1 NKAP MAGEA3 PDE3A KANK4 SOCS3 LZTS3 PROCA1 FGFBP1 HES2 BMF TSPAN12 P3H2 ITPKB RPRD1A TSPYL1 RNF24 RAP2C DLG5 WIPF1 CALD1 IDE MYLK3 FZD4 WNT7B TWIST1 FBXO2 TYMP C1R RGS16 B3GNT9 TAS1R3 TSC22D3 ETS2 IGFBP7 NOTUM TNNC1 KAZALD1 PRKAB2 HELZ2 CAPG DUSP1 H1F0 MMP28 MGLL MN1 SCARA5 LINC00602 ALDH3A1 FAM43A SPON2 SALL4 KRT10 KCTD12 NKX3-2 JUN ADAMTS5 KCNN4

Supplementary Table 3 Primers used in the present study

Gene	Primer sequence
**qRT-PCR**	
PDK4	forward: 5’- CAAGATGCCTTTGAGTGTTCAA -3’
	reverse: 5’- GGTCTTCTTTTCCCAAGACAAC -3’
Pre-PDK4	forward: 5’- CCAGGCATGGTGGCACACAC -3’
	reverse: 5’- GCTGGAGTGCAGTGGGACAATC -3’
PDK1	forward: 5’- AACCGACACAATGATGTCATTC -3’
	reverse: 5’- ATGCGACTCATGTAGAATCGAT-3’
PDK2	forward: 5’- CTCTACGCCAAGTACTTCCAG -3’
	reverse: 5’- GCCTTGAGATAGATGACAGCAT -3’
PDK3	forward: 5’- CTGCTGTTAAAACCCTCGTTAC-3’
	reverse: 5’- CACTCCTTCCATGGAATACAGT -3’
GAPDH	forward: 5’- CAGAACATCATCCCTGCCTCTAC-3’
	reverse: 5’- TTGAAGTCAGAGGAGACCACCTG-3’
Mettl3	forward: 5’- CTATCTCCTGGCACTCGCAAGA -3’
	reverse: 5’- GCTTGAACCGTGCAACCACATC-3’
ALKBH5	forward: 5’- CCAGCTATGCTTCAGATCGCCT -3’
	reverse: 5’- GGTTCTCTTCCTTGTCCATCTCC-3’
HPRT	forward: 5’- TGACACTGGCAAAACAATGCA-3’
	reverse: 5’- GGTCCTTTTCACCAGCAAGCT-3’
MALAT1	forward: 5’- GCTTGGCTTCTTCTGGACTCA-3’
	reverse: 5’- TCGCGAGCTTCACCATGA -3’
Firefy-Luc	forward: 5’- GGTACTGTTGGTAAAGCCAC-3’
	reverse: 5’- CTCTTCATAGCCTTATGCAG-3’
Renilla-Luc	forward: 5’- CAATGGGCAGGTGTCCACTC -3’
	reverse: 5’- GTTCTGGATCATAAACTTTC -3’
ETS1	forward:5’- TTGAAAGCATAGAGAGCTACGA-3’
	reverse:5’- CTCTGAGTCGAAGCTGTCATAG-3’
FOXA1	forward:5’- GTTCTCCATCAACAACCTCATG-3’
	reverse:5’- TATTGCAGTGCCTGTTCGTAT-3’
NRF1	forward:5’- TGCCGTGGCTGATGGAGAGG-3’
	reverse:5’- GATGCTTGCGTCGTCTGGATGG-3’
PAX5	forward:5’- CTTCCAGTCACAGCATAGTGTC-3’
	reverse:5’- CTCCTGAATACCTTCGTCTCTC-3’
STAT4	forward:5’- CTCTGCCATTCGCTGACATCCTG-3’
	reverse:5’- CTTCGCAAGGCTGAGAGCTGTAG-3’
TBP	forward:5’- CCGGAATCCCTATCTTTAGTCC-3’
	reverse:5’- GCCTTTGTTGCTCTTCCAAAAT-3’
TP53	forward:5’- TTCCTGAAAACAACGTTCTGTC-3’
	reverse:5’- AACCATTGTTCAATATCGTCCG-3’
YY1	forward:5’- CCCACGGTCCCAGAGTCCAC-3’

	reverse:5’- AAAGCGTTTCCCACAGCCTTCG-3’
qPCR-SELECT	forward:5’- ATGCAGCGACTCAGCCTCTG-3’<br>reverse:5’- TAGCCAGTACCGTAGTGCGTG-3’
**Mutation plasmid**	**Primer sequence**
PDK4/5’UTR-Mut1	forward: 5’- GTCCTCGCCCGGCCGCGCCGCGCCC -3’<br>reverse: 5’- CACGTGGCATTCGTTCAGAC -3’
PDK4/3’UTR-Mut1	forward: 5’- CGCTAGCAGAGGGcCACTCAGGACAC -3’<br>reverse: 5’- GTGTCCTGAGTGGCCCTCTGCTAGCG -3’
PDK4/3’UTR-Mut2	forward: 5’- GGGACACTCAGGcCACTTTACGGGATC-3’<br>reverse: 5’- GATCCCGTAAAGTGGCCTGAGTGTCCC-3’
PDK4/3’UTR-Mut3	forward: 5’- GCAAATAGCCTGGCCGTAAGATTTCTC-3’<br>reverse: 5’- GAGAATCTCTTACGGCCAGGCTATTTGC -3’
**SELECT**	**Primer sequence**
PDK4-X1	forward: 5’- tagccagtaccgtagtgcgtg CCAGCCAGCGCACCTGCAGTCCTCG -3’<br>reverse: 5phos/GAGCCTGGTTCCGAGGGGGCGCGGC cagaggctgagtcgctgcat -3’
PDK4-N1	forward: 5’- tagccagtaccgtagtgcgtg CCAGCCAGCGCACCTG -3’<br>reverse: 5’- 5phos/GAGCCTGGTTCCGAGGGGGCGC cagaggctgagtcgctgcat -3’
PDK4-X2	forward: 5’- tagccagtaccgtagtgcgtg CCAAAGAACCTGGCAAAAGAAGTGG -3’<br>reverse: 5’- 5phos/CACTTTGATCCCGTAAAGTGTCCTG cagaggctgagtcgctgcat -3’
PDK4-N2	forward: 5’- tagccagtaccgtagtgcgtg CCAAAGAACCTGGCAAAAGAAGTGG -3’<br>reverse: 5’- 5phos/GACCCACTTTGATCCCGcagaggctgagtcgctgcat -3’
PDK4-X3	forward: 5’- tagccagtaccgtagtgcgtg GCAAATAGCCTGGACG -3’<br>reverse: 5’- 5phos/GCATGCTGGCATGAGAATCTCcagaggctgagtcgctgcat -3’
PDK4-N3	forward: 5’- tagccagtaccgtagtgcgtg GCAAATAGCCTGGACG -3’<br>reverse: 5’- 5phos/GCATGCTGGCATGAGAATCTCcagaggctgagtcgctgcat -3’

Supplementary References

1. Wang X*, et al.* N(6)-methyladenosine Modulates Messenger RNA Translation Efficiency. *Cell* **161**, 1388-1399 (2015).
