# Supplementary material for: N6-methyladenosine regulates glycolysis of cancer cells through PDK4
Source: Nat Commun. 2020 May 22;11:2578. doi: 10.1038/s41467-020-16306-5 (PMC7244544; doi:10.1038/s41467-020-16306-5)
Supplement: Supplementary file 3 — Description of Additional Supplementary Files [file 41467_2020_16306_MOESM3_ESM.pdf]

**Description of Additional Supplementary Files**

**File Name:** Supplementary Data 1

**Description:** The Supplementary Data 1 shows the difference genes variated greater than 2.0-fold variation (*p*<0.05 by random permutation test) between wild type and *Mettl3*$^{Mut/-}$ HeLa cells

**File Name:** Supplementary Data 2

**Description:** The Supplementary Data 2 shows the overlap of genes among which were involved in glucose metabolism, variated greater than 2.0-fold variation (*p*<0.05 by random permutation test) between wild type and *Mettl3*$^{Mut/-}$ HeLa cells , and modified by $m^6A$ in HeLa cells modification is more than 3 times greater than that in the input.
