# Supplementary material for: N6-methyladenosine regulates glycolysis of cancer cells through PDK4
Source: Nat Commun. 2020 May 22;11:2578. doi: 10.1038/s41467-020-16306-5 (PMC7244544; doi:10.1038/s41467-020-16306-5)
Supplement: Supplementary file 7 — Source Data [file 41467_2020_16306_MOESM7_ESM.pdf]

Source data

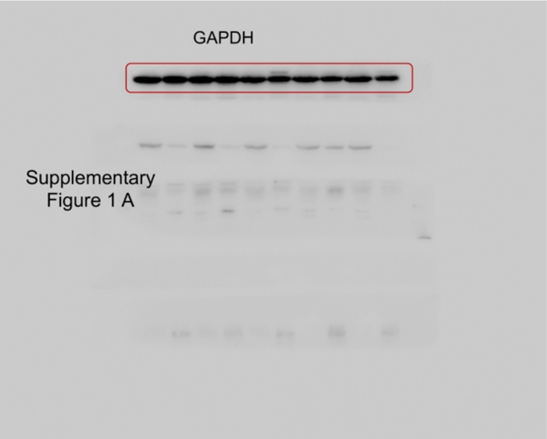
GAPDH
Supplementary
Figure 1 A

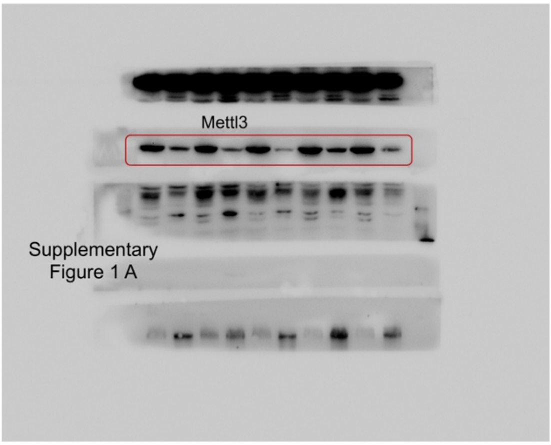
Mettl3
Supplementary
Figure 1 A

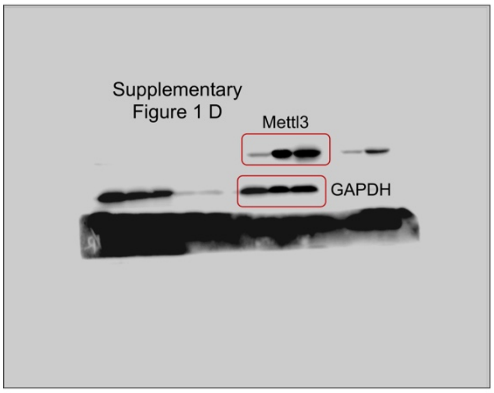
Supplementary
Figure 1 D
Mettl3
GAPDH

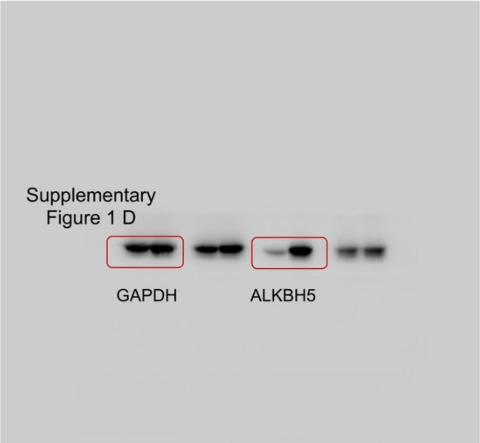
Supplementary
Figure 1 D
GAPDH
ALKBH5

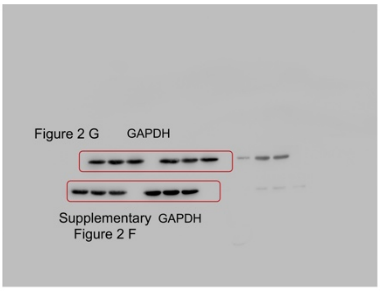
Figure 2 G
GAPDH
Supplementary
Figure 2 F
GAPDH

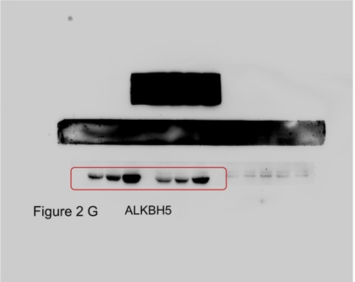
Figure 2 G
ALKBH5

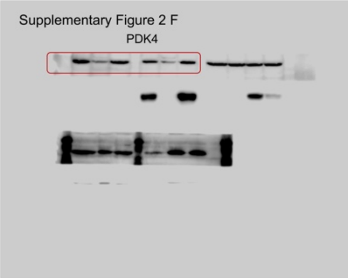
Supplementary Figure 2 F
PDK4

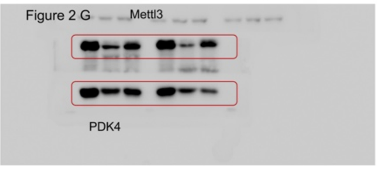
Figure 2 G
Mettl3
PDK4

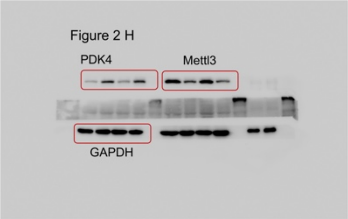
Figure 2 H
PDK4
Mettl3
GAPDH

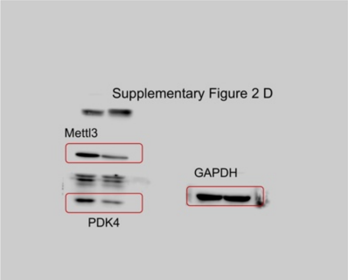
Supplementary Figure 2 D
Mettl3
GAPDH
PDK4

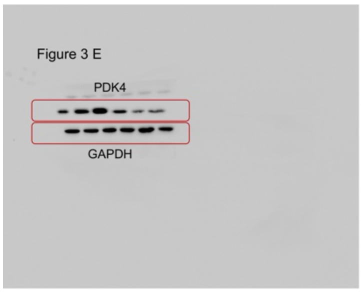
Figure 3 E
PDK4
GAPDH

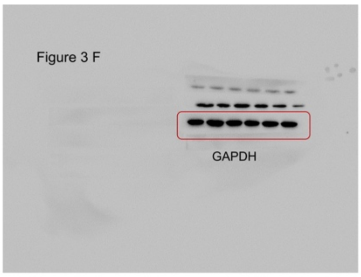
Figure 3 F
GAPDH

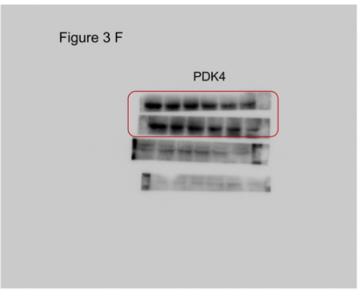
Figure 3 F
PDK4

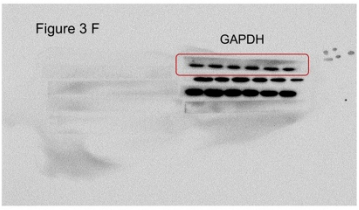
Figure 3 F
GAPDH

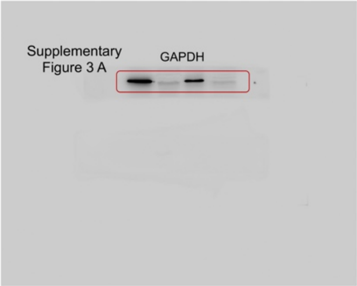
Supplementary
Figure 3 A
GAPDH

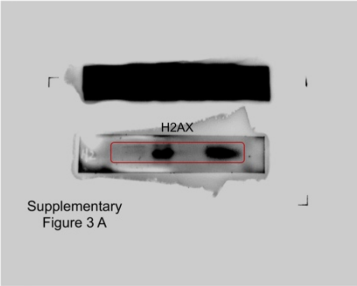
H2AX
Supplementary
Figure 3 A

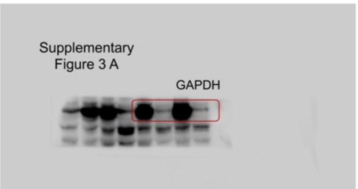
Supplementary
Figure 3 A
GAPDH

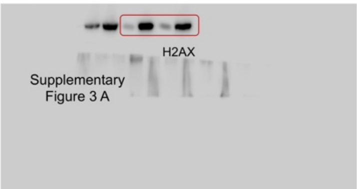
H2AX
Supplementary
Figure 3 A

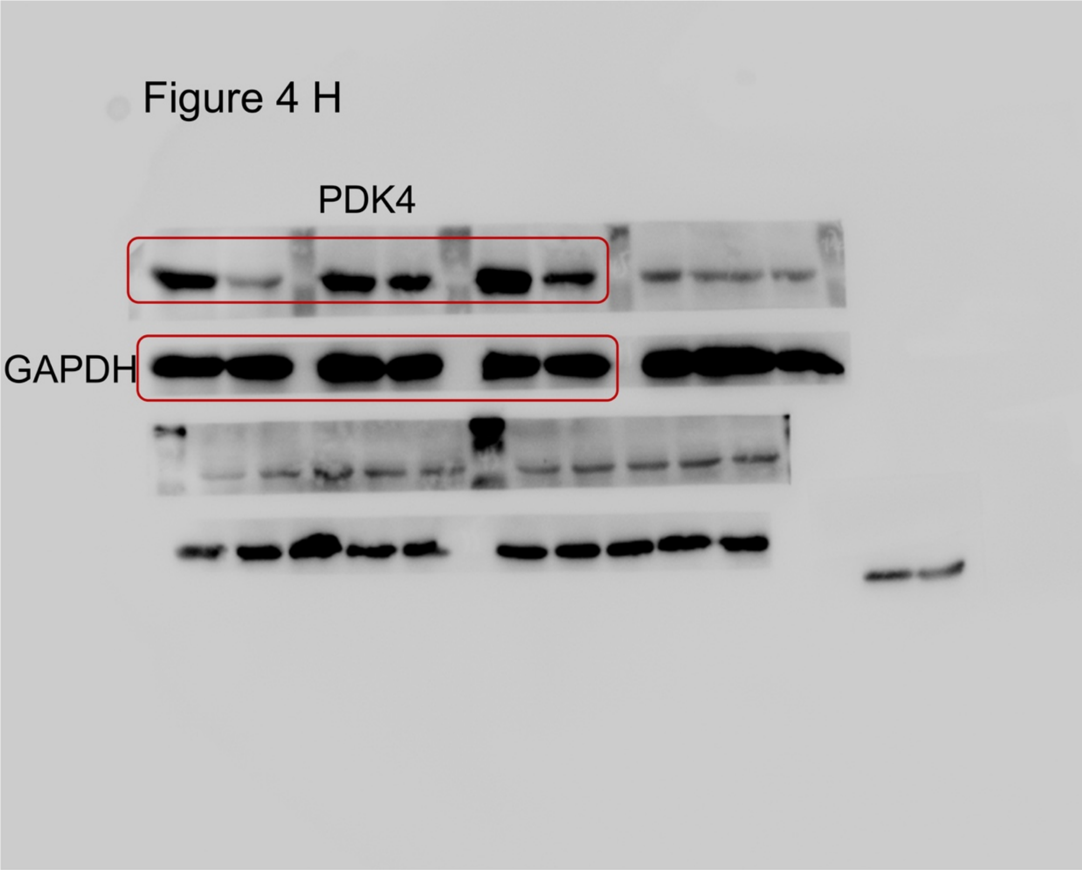
Figure 4 H
PDK4
GAPDH

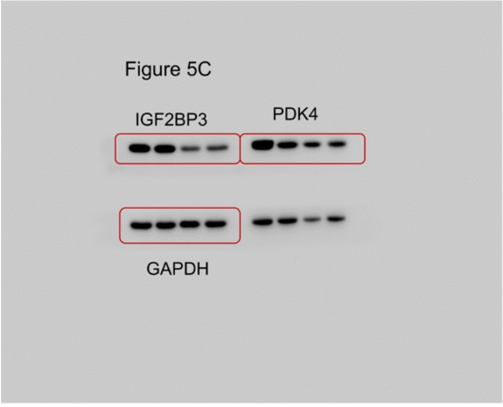
Figure 5C
IGF2BP3
PDK4
GAPDH

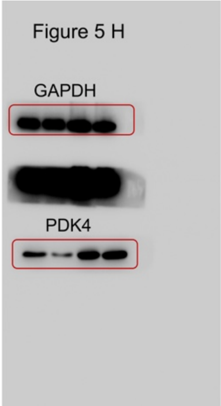
Figure 5 H
GAPDH
PDK4

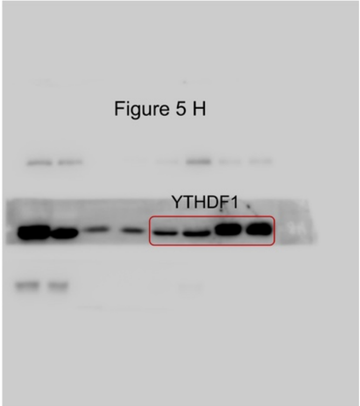
Figure 5 H
YTHDF1

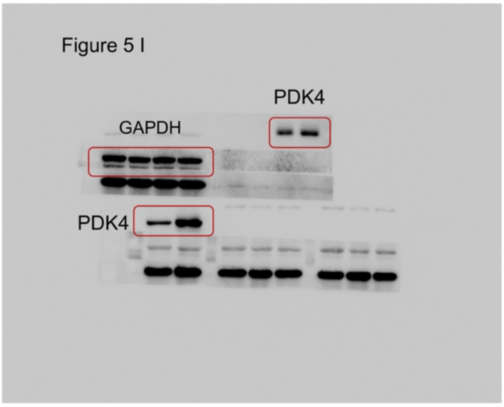
Figure 5 I
PDK4
GAPDH
PDK4

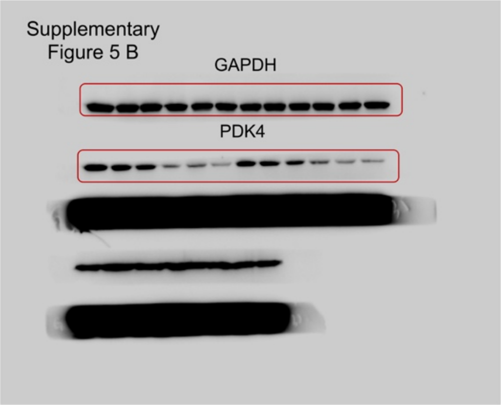
Supplementary
Figure 5 B
GAPDH
PDK4

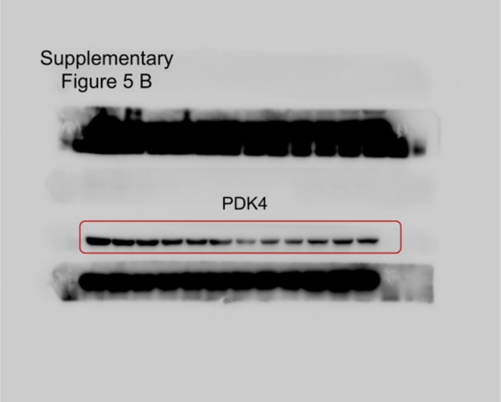
Supplementary
Figure 5 B
PDK4

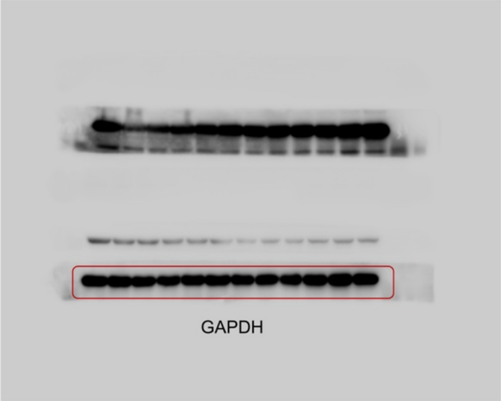
GAPDH

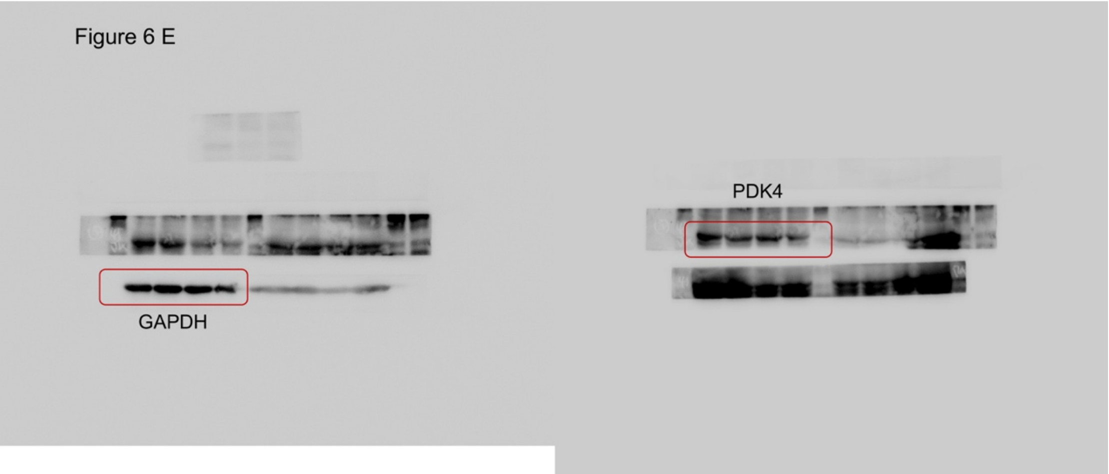
Figure 6 E
GAPDH
PDK4

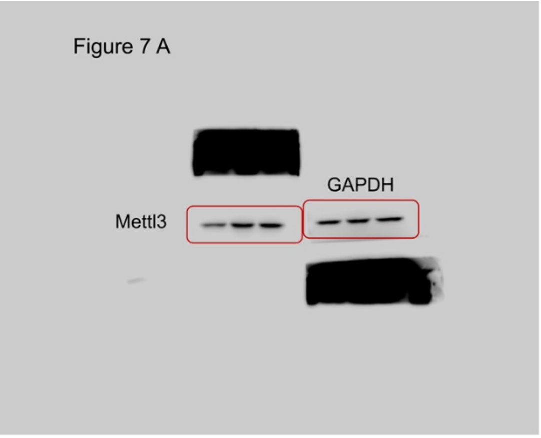
Figure 7 A
GAPDH
Mettl3

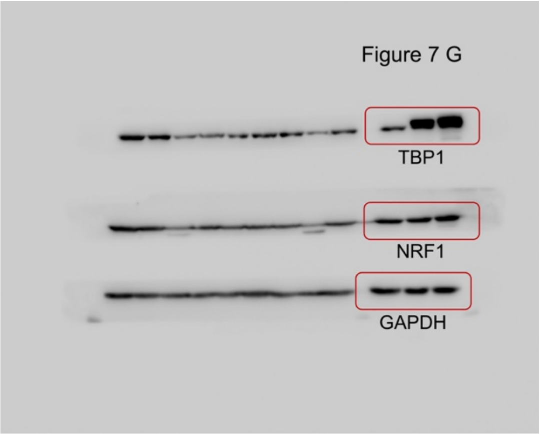
Figure 7 G
TBP1
NRF1
GAPDH

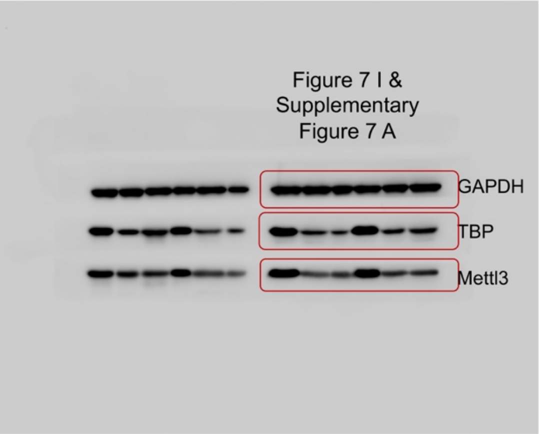
Figure 7 I &
Supplementary
Figure 7 A
GAPDH
TBP
Mettl3

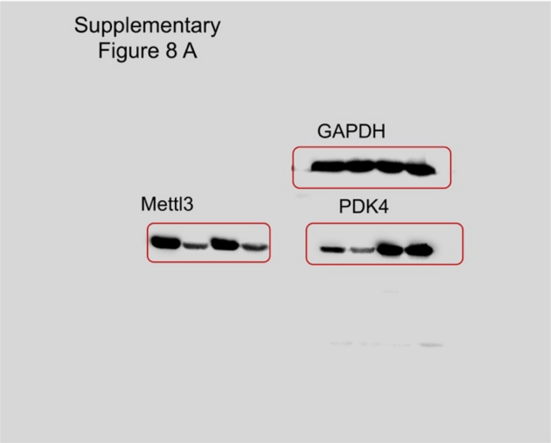
Supplementary
Figure 8 A
GAPDH
Mettl3
PDK4

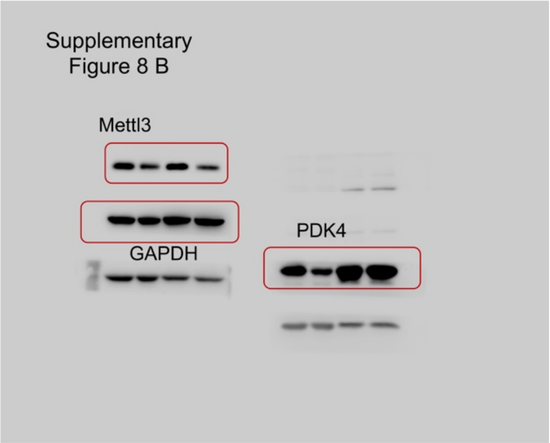
Supplementary
Figure 8 B
Mettl3
PDK4
GAPDH

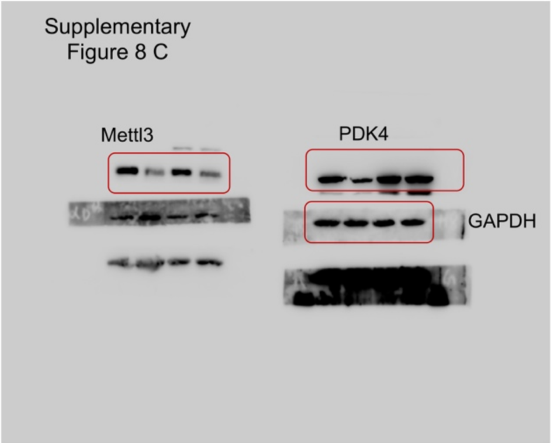
Supplementary
Figure 8 C
Mettl3
PDK4
GAPDH
